# Cytoplasmic Actin Is an Extracellular Insect Immune Factor which Is Secreted upon Immune Challenge and Mediates Phagocytosis and Direct Killing of Bacteria, and Is a *Plasmodium* Antagonist

**DOI:** 10.1371/journal.ppat.1004631

**Published:** 2015-02-06

**Authors:** Simone L. Sandiford, Yuemei Dong, Andrew Pike, Benjamin J. Blumberg, Ana C. Bahia, George Dimopoulos

**Affiliations:** W. Harry Feinstone Department of Molecular Microbiology and Immunology, Bloomberg School of Public Health, Johns Hopkins University, Baltimore, Maryland, United States of America; University of Pennsylvania School of Medicine, UNITED STATES

## Abstract

Actin is a highly versatile, abundant, and conserved protein, with functions in a variety of intracellular processes. Here, we describe a novel role for insect cytoplasmic actin as an extracellular pathogen recognition factor that mediates antibacterial defense. Insect actins are secreted from cells upon immune challenge through an exosome-independent pathway. *Anopheles gambiae* actin interacts with the extracellular MD2-like immune factor AgMDL1, and binds to the surfaces of bacteria, mediating their phagocytosis and direct killing. Globular and filamentous actins display distinct functions as extracellular immune factors, and mosquito actin is a *Plasmodium* infection antagonist.

## Author Summary

Actin is one of the best studied, evolutionary conserved and most abundant intracellular proteins. Actin can exists in globular and filamentous functionally distinct forms, and is involved in a variety of biological processes, such as muscle contraction, cell motility, cell division, vesicle and organelle movement, endocytosis, and cell signaling. Here we show a novel function of insect cytoplasmic actin, as an extracellular immune factor. Actin is externalized by insect immune competent cells upon immune challenge with bacteria or bacterial surface components, and once externalized, actin binds with high affinity to the surface of bacteria. A functional role of actin’s interaction with bacteria is to mediate their killing through either phagocytosis or direct antibacterial action. The globular and filamentous forms of actins appear to play distinct functions as extracellular immune factors. Actin also plays a role as a *Plasmodium* antagonist as it limits parasite infection of the mosquito gut tissue.

## Introduction

Actin is one of the evolutionary most conserved and abundant proteins found in eukaryotic cells, and exists in globular and filamentous functionally distinct forms. In vertebrates, three main actin isoforms have been described, alpha, beta and gamma; alpha actins are found in muscle tissues whereas beta and gamma actins coexist and comprise the cytoskeleton [1, 2]. Globular and filamentous actins interact with numerous proteins, and are involved in a variety of vital biological processes including muscle contraction, cell motility, cell division, vesicle and organelle movement, endocytosis, and cell signaling [1, 2]. Moreover, actin plays an essential role in immunity as all phagocytic processes involve reorganization of the actin cytoskeleton [3]. Interestingly, filamentous actin has recently been identified as the ligand for a receptor that recognizes damaged and dying cells [4, 5].

*Anopheles gambiae* actins are encoded by multigene families and are classified based on homology with *Drosophila melongaster* actins. The five *An. gambiae* actin genes belong to three functional groups: cytoskeletal, adult muscle and larval muscle actins. Similar to *D. melanogaster* the constitutively expressed cytoplasmic actin (actin 5C; 651A-C) has three alternative promoters leading to the production of three identical cytoplasmic actin isoforms [6]. Proteomic analyses have detected actins in the hemocyte-containing hemolymph fractions of various mosquito species, and actin 5C was specifically identified in the hemolymph of *D. melanogaster* larvae that had been challenged with either *Micrococcus luteus*, *Saccharomyces cerevisiae* or lipopolysaccharide (LPS) but not in hemolymph of naïve larvae. Challenge of *D. melanogaster* larvae with LPS for 4 hr resulted in a 10-fold increase of actin 5C in the hemolymph, while sterile injury did not lead to detectable amounts of the protein [7, 8, 9, 10]. Actin has also been found in the supernatant fraction of HIV-infected human macrophage cells [11, 12]. However, the functional role and significance of extracellular actins has not been previously addressed.

The insect innate immune system is activated through recognition of pathogen associated molecular patterns (PAMPs) by pattern recognition receptors (PRRs). The PRRs can either directly mediate anti-pathogen defenses, such as phagocytosis, or indirectly modulate defenses through the activation of immune pathways, such as the Toll, Immune Deficiency (Imd) and the Janus kinase-signal transducers and activators of transcription (JAK-STAT) pathways. The innate immune system of mosquito disease vectors has gained increasing attention because of their implication in regulating vector competence for human pathogens [13]. Several mosquito PRRs that mediate the killing of pathogens, including *Plasmodium*, have been characterized [14]. Significant overlap exists between the mosquitoes’ antibacterial and anti-*Plasmodium* defenses and PRRs have been shown to interact with, and mediate killing of, both pathogen classes [15, 16, 17]. The high degree of conservation between the innate immune systems of mammals and invertebrates has greatly facilitated its functional dissection since many discoveries in either model organism are relevant across phyla.

A conserved immune factor and PRR is the *An. gambiae* homolog of the vertebrate myeloid differentiation factor-2 (MD-2) protein, AgMDL1. In vertebrates, MD-2 is necessary for LPS-mediated Toll pathway activation through a complex with the TLR4 receptor [18, 19, 20]. Although multiple proteins containing the MD-2 related lipid recognition (ML) domain have been identified in various insect species, their role in immunity is still not well understood. In *D. melanogaster*, for instance, only some ML domain containing proteins have been shown to interact with components of the bacterial cell wall [21]. We have previously shown that the *An. gambiae* AgMDL1 is important for adult mosquito resistance to systemic bacterial challenge and that it is an antagonist of the human malaria parasite *Plasmodium falciparum* [17].

Here we identified the ubiquitous protein actin 5C as a novel extracellular immune factor through its interaction with bacterial surfaces and the PRR AgMDL1. We further show that actin 5C mediates antibacterial defense as an extracellular PRR through phagocytosis and direct killing. Actin 5C is a *Plasmodium* antagonist at the stage of mosquito midgut infection. Globular and filamentous actins appear to play distinct roles in insect immunity.

## Results

### Actin is a bacteria binding protein that interacts with the *An. gambiae* MD2 homolog AgMDL1

Since many of the known mosquito PRRs have been shown to interact with bacterial surfaces we employed a bacteria binding assay with mosquito protein extracts in conjunction with an isobaric tags for relative and absolute quantitation (iTRAQ) proteomic approach to identify additional bacteria-binding PRRs of the mosquito’s immune surveillance system [15, 22, 23, 24]. We used this quantitative proteomic approach to identify putative PRRs that bind to an *Enterobacter* bacterium with high affinity. Bacteria were incubated with soluble lysates from either the immune competent Sua5B cell line, adult female *An. gambiae* mosquitoes or larval extracts, and bacterial binding proteins were subsequently eluted using increasingly stringent sodium chloride washes. The proteome of the 0.5M NaCl eluate was analyzed by iTRAQ, revealing actin 5C (AGAP000651), along with other proteins, as a high affinity bacterial binding protein. In a parallel study, aimed at dissecting the function of the *An. gambiae* immune factor AgMDL1 (AGAP012352) in the defense against pathogens, we conducted a yeast two-hybrid screen to identify potential AgMDL1 binding partners. An *An. gambiae* Sua5B cell line cDNA library was used as the prey and the full-length AgMDL1 was used as the bait. Screening of 6.5 million diploids generated 17 clones that grew on the low stringency double drop out medium. To identify high affinity protein-protein interactions and to minimize the probability of false positives, positive clones from the yeast two-hybrid screen were then re-screened using higher stringency media. Two of the 5 identified clones encoded actin 5C. To confirm this interaction, a His-tagged pull-down assay was performed using recombinant AgMDL1 as bait against a Sua5B cell line soluble lysate extract. In agreement with the yeast two-hybrid data, AgMDL1 binds to actin present in the Sua5B cell lysate extract (S1 Fig.).

### Actins display diverse developmental stage- and tissue-specific expression

**Fig 1:**
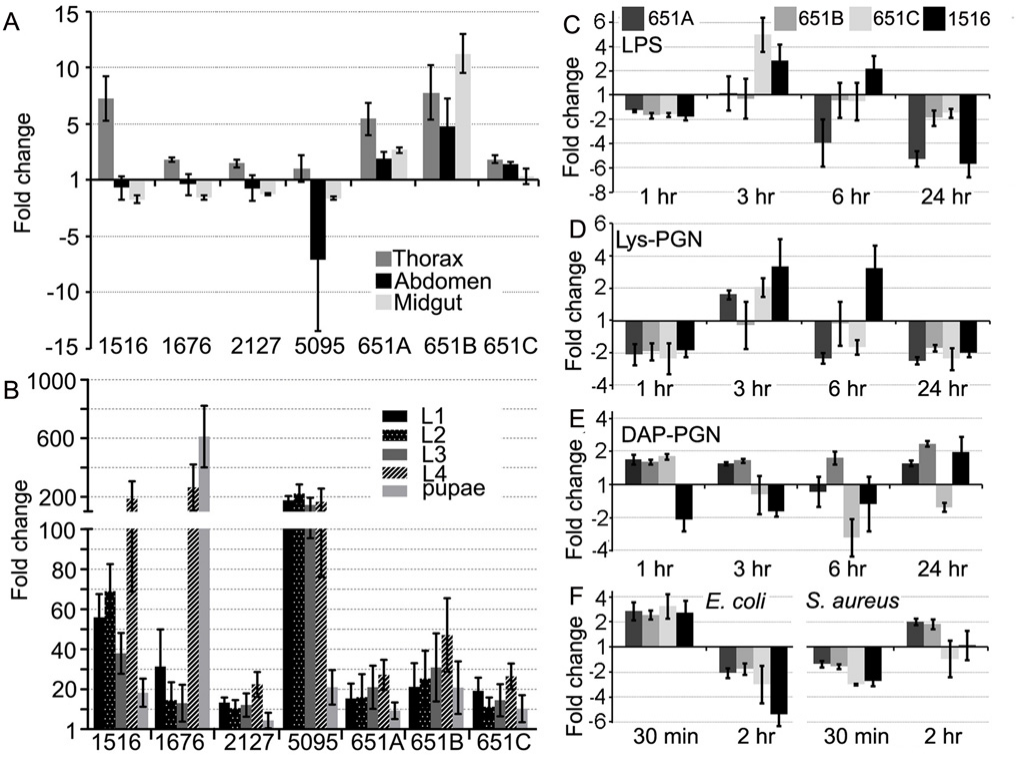
*An. gambiae* actins display tissue, developmental-specific, and immune-responsive expression. Transcript abundance of all *An. gambiae actin* genes in the (A) thorax, abdomen, and midgut adult tissues or (B) at distinct developmental stages compared to the adult female mosquito and normalized using the *An. gambiae* ribosomal *S7* gene. Actin 5C (651 A, B, C); larval muscle actin (5095); adult muscle actin (1516); minor actins (1676) and (2127). Expression of actin 5C (651 A, B, C) and adult muscle actin (1516) in *An. gambiae* Sua5B cells after challenge with (C) LPS (10 μg/mL) (D) Lys-PGN (20 μg/mL) or (E) DAP-PGN (1 μg/mL) for 1,3,6 and 24 hr or (F) live *E. coli* and *S. aureus* (MOI 100) for 30 min or 2 hr, compared to non-challenged cells and normalized using the *An. gambiae S7* gene.

To gain insight on the functional diversity of the five *An. gambiae* actins we determined their developmental and tissue-specific transcript abundance. Their expression patterns are diverse, suggesting specialized functions in different developmental stages and tissues, similar to what has been previously reported for *D. melanogaster* actins [25, 26]. Transcripts of the major adult muscle *actin* (1516) for example display a greater than 5-fold higher abundance in the flight muscle-containing thorax when compared to the whole female (Fig. 1A). As expected, the larval muscle *actin* (5095) is down regulated in adult female tissues compared to larval developmental stages. Interestingly, whereas all three isoforms of the constitutively expressed cytoplasmic *actin 5C* (651A–C) display similar transcript abundance patterns during development (Fig. 1B), they exhibit differential expression levels in adult tissues suggesting non-redundant roles in various physiological processes.

### Actins display immune-responsive expression

Actin 5C’s association with the bacterial surface and with the immune factor AgMDL1 suggested a possible function as an immune factor. Genes playing roles in the insect immune system are frequently transcriptionally regulated by immune challenge. Exposure of *An. gambiae* Sua5B cells to the immune elicitors and major bacterial cell wall components LPS (10 μg/mL of an LPS-cocktail containing combinations of *Escherichia coli (E.c.), Klebsiella pneumonia (K.p.)* and *Pseudomonas aeruginosa (P.a.)*: hereafter indicated as LPS), *E. coli*-derived DAP type peptidoglycan (DAP-PGN; 1 μg/mL) or *Staphylococcus aureus*-derived lysine type peptidoglycan (Lys-PGN; 20 μg/mL), or live bacteria (*E. coli* or *Staphylococcus aureus*, at a multiplicity of infection (MOI) of 100) induced changes in the transcript abundance of all *actins* that had detectable expression levels in the cell line (Fig. 1C–F). The three different isoforms of *actin 5C* displayed differential changes in transcript abundance upon the immune challenges, suggesting that their functions are not completely redundant. Interestingly, the LPS and *S. aureus*-derived Lys-PGN-induced more similar changes in transcript abundance than those induced by *E. coli*-derived DAP-PGN (Fig. 1C–E). Incubation of cells with either live *E. coli* or *S. aureus* revealed opposite changes in transcript abundance (Fig. 1F). These data suggest that *actin* transcription is regulated through multiple pathways and that additional bacterial surface components can stimulate *actin* transcription. The infection responsive changes in *actin* transcript abundance further suggested functional roles in immune-related processes.

### Actin is secreted by immune competent insect cell lines upon immune challenge

**Fig 2:**
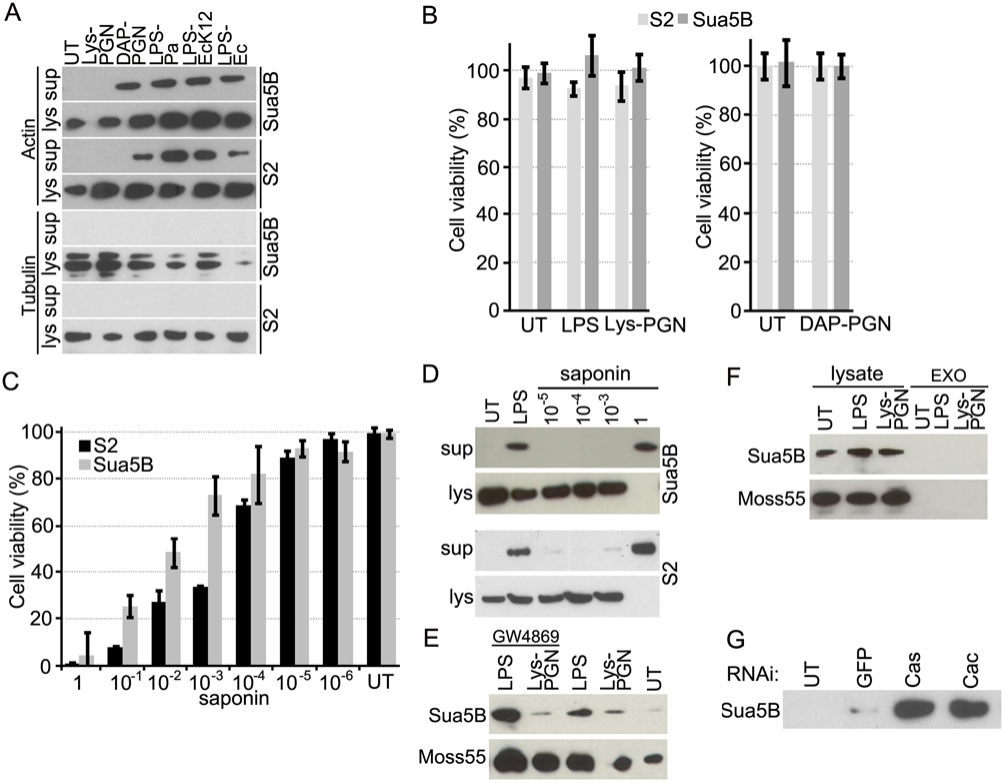
Actin is secreted into the cell culture supernatant fraction upon immune challenge via an exosome independent mechanism that is regulated by immune pathways. (A) Immune-challenged (LPS-Pa, LPS-EcK12, LPS-Ec; 10 μg/mL Lys-PGN; 20 μg/mL, or DAP-PGN; 1 μg/mL) Sua5B (*An. gambiae*), and S2 (*D. melongaster*) insect cell supernatants and soluble lysate fractions examined for the presence of actin (upper panel) or tubulin (lower panel). (B) Viability of Sua5B and S2 cell lines after treatment with LPS-Pa (10 μg/mL), Lys-PGN (20 μg/mL) or DAP-PGN (1 μg/mL) for 24 hr was determined using the Cell Titer Fluor Cell Viability Assay. (C) Cell viability of Sua5B and S2 cells after saponin (1–10^−6^%)-induced cell lysis and (D) the amount of actin released into the supernatant was determined for Sua5B (upper panel) and S2 cells (lower panel) at varying saponin concentrations (10^−5^, 10^−4^, 10^−3^ and 1%). (E) Supernatant fractions of Sua5B and Moss55 cells challenged with LPS (10 μg/mL) or Lys-PGN (20 μg/mL) for 24 hr in the presence of 5 μM GW4869 or DMSO (control) and probed for actin. (F) Exosomes (EXO) and lysate fractions isolated from LPS and Lys-PGN stimulated Sua5B or Moss55 cells analyzed for actin. (G) Supernatant fraction of *caspar* and *cactus* silenced Sua5B cells probed for the presence of actin.

The interaction of *An. gambiae* actin 5C with the secreted immune factor AgMDL1 in addition to the infection-responsive expression of *actin* transcripts, and previous reports demonstrating the presence of actin in the mosquito hemolymph and the immune-challenged *D. melanogaster* larval hemolymph [8, 9], lead us to investigate whether actin is secreted by immune competent insect cell lines upon immune challenge. A 24 hr exposure of *An. gambiae* Sua5B and *D. melanogaster* S2 cells with 20 μg/mL of the Lys-PGN did not induce actin secretion, while treatment with 1 μg/mL of the DAP-PGN, also a potent Imd pathway activator [27], did (Fig. 2A). Treatment of both cell types with 10μg/mL of either *P. aeruginosa* (serotype 10)-derived LPS (LPS-Pa), an ultra-pure preparation of the *E. coli* K12—derived LPS (LPS-EcK12) or an *E. coli* O11:B4—derived LPS (LPS-Ec) induced actin secretion in the cell line supernatant. We also tested additional cell lines from different mosquito species for either LPS- or Lys-PGN-mediated actin secretion and showed that *Anopheles stephensi* MSQ43, *Aedes aegypti* Aag-2 and the *Aedes albopictus* C6/36 cell lines also specifically externalized actin upon LPS challenge but not Lys-PGN challenge (S2 Fig.). The extent of actin externalization varied among the different cell lines. The *An. gambiae* Moss55 cells constitutively externalized actin into the supernatant, and LPS stimulation did not produce an additive effect. Cell Titer Fluor (Promega) cell viability assays and microscopy observations showed that LPS, Lys-PGN or DAP-PGN treatment did not cause cell death or lysis, which could result in the release of actin into the culture supernatant (Fig. 2B). Controlled lysis of Sua5B and S2 cells, through treatment with saponin concentrations of 10^−5^, 10^−4^ and 10^−3^%, corresponding to approximately 7, 18 and 27% cell mortality for Sua5B cells, and 10, 31 and 67% cell mortality for S2 cells, did not result in a comparable amount of externalized actin when compared to that of LPS treated cells that did not cause any detectable mortality (Fig. 2C, D). LPS- or Lys-PGN-challenge of other mosquito cell lines did also not result in increased mortality (S2B Fig.). A large amount of externalized actin was only detected in the supernatant fraction of cells treated with 1% saponin which results in 96% and 99% cell death of Sua5B and S2 cells, respectively. To provide further evidence that actin is specifically externalized upon immune challenge, we investigated whether alpha tubulin, another abundant cytosolic protein is detected in the supernatant fraction of cells after LPS, DAP-PGN or Lys-PGN treatment. Western blot analysis confirms that tubulin is not present in the supernatant fraction after exposure to the immune elicitors (Fig. 2A). Accordingly, here we show that insect immune competent cell lines externalize actin in response to immune challenge, and this effect is not a result of cell mortality and lysis.

### Actin is secreted through an exosome-independent mechanism

Proteins that do not contain signal peptides are secreted through unconventional secretory pathways, and may either cross the plasma membrane directly or by association with secretory vesicles that fuse with the membrane for release into the extracellular environment [28, 29, 30]. However, while human beta actin has been shown to be secreted through exosomes [11], disruption of exosome formation through treatment of both Sua5B and Moss55 cells with the nSMase inhibitor GW4869 resulted in an increased amount of externalized actin upon LPS stimulation (Fig. 2E) [31]. This was possibly a result of the general up-regulation of an alternative secretion pathway upon inhibition of exosome-mediated secretion. Furthermore, exosomes isolated from both cell lines did not contain actin (Fig. 2F). Other immune factors such as insect prophenoloxidases and the vertebrate interleukin 1 also lack signal peptides, and their route of externalization remains unknown [32].

### Actin secretion is regulated by the Imd and Toll pathways

The mosquito and fly antibacterial defenses are to a significant extent regulated by the Toll and Imd immune signaling pathways which are known to be activated by bacterial surface components including PGN [20, 33, 34]. We therefore explored whether activation of any of these two immune signaling pathways could regulate actin secretion. Activation of the Toll and Imd pathways, by silencing their negative regulators *cactus* and *caspar*, respectively, in Sua5B cells according to established methodology [35], without immune challenge resulted in actin secretion into the cell line supernatant fraction (Fig. 2G). Hence, these results corroborate an immune function of actin externalization and implicate two major immune signaling pathways in this process.

### Interaction of actin with Gram-positive and Gram-negative bacteria is enhanced in the presence of AgMDL1

**Fig 3:**
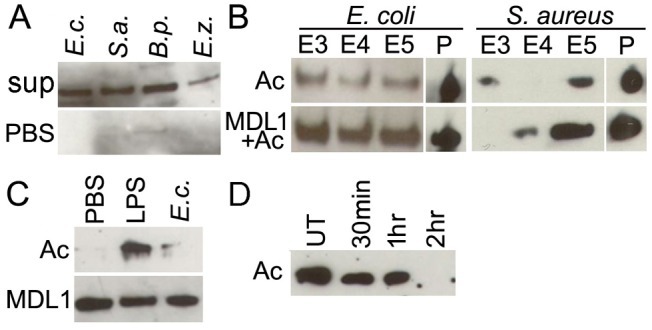
Actin binds to the surface of different bacteria and is externalized into the mosquito hemolymph upon immune challenge. Analysis of actin content in the (A) 0.3 M NaCl-eluted fraction of Gram-negative (*E. coli*, *E.c; Esp_Z, E.z.*) and Gram-positive (*S. aureus*, *S. a.; B. pumilus B. p.*) bacterial species after incubation with immune-challenged cell line supernatants, or (B) 0.3–0.5 M NaCl (E3–E5)-eluted fractions and pellets (P) of Gram-negative (*E. coli*) and Gram-positive (*S. aureus*) bacteria incubated with recombinant actin alone (Ac), or together with AgMDL1 (Ac+MDL1). (C) Mosquito hemolymph probed for actin (Ac) and AgMDL1 (MDL1) after challenge with PBS, *E. coli* (*E.c.* OD 2.8) or LPS-Pa (100 ng) for 4 hr. (D) Actin content in Moss55 cell supernatant after incubation with *E. coli (E.c.* MOI 1000) for 30 min, 1 hr, or 2 hr or untreated (UT).

To determine whether actin can interact with bacterial surfaces other than *Enterobacter*, we performed bacterial binding assays with LPS-stimulated insect cell line supernatants according to established methodology [15]. Incubation of 500 μL (OD 3.2) of gram-positive (*Staphylococcus aureus* and *Bacillus pumilus*,) and gram-negative (*E. coli* and *Enterobacter Esp_Z*) bacteria (OD 3.2) with 4 mL LPS-stimulated insect cell line supernatants and then eluting proteins bound to the bacteria with increasingly stringent NaCl washes revealed that actin interacts with both gram-positive and negative bacterial species (Fig. 3A). After incubation of either *E. coli* or *S. aureus* (25 μL, OD 3.2) with recombinant actin 5C (8 μg), with or without AgMDL1 (8 μg), actin was detected regardless of AgMDL1 in the high-salt eluate fractions as well as in the bacterial pellet after a series of increasingly stringent salt washes, suggesting that actin can engage in an AgMDL1-independent high-affinity interaction with the bacterial surface (Fig. 3B). The interaction of actin 5C with bacterial surfaces was however augmented in the presence of AgMDL1, indicating a possible functional role for this complex. As we describe below, we also confirmed the interaction of actin and AgMDL1 with FITC labeled *E. coli* using confocal microscopy immunofluorescence assays.

### Bacterial challenge of adult mosquitoes results in secretion of actin into the hemolymph and subsequent sequestration by the bacteria

We then examined if secretion of actin is a physiologically relevant process in adult mosquitoes by injecting LPS (100ng) into female *An. gambiae* mosquitoes and probing the hemocyte depleted hemolymph after 4 hr for the presence of actin. In support of our cell line-based experiments, we detected actin in the LPS-challenged mosquito hemolymph fraction (Fig. 3C), similarly to what had been observed with LPS-challenged *D. melanogaster* larvae [10]. Furthermore, injection of adult mosquitoes with approximately 1.9 × 10^5^ live *E. coli* resulted in the detection of only approximately 10% of the actin found in the hemolymph of LPS-injected mosquitoes, at 4 hr after challenge, as determined by densitometric analysis of the western blot, presumably because actin was sequestered by binding to the bacteria, as we have shown in the *in vitro* experiments (Fig. 3A, B). To confirm this hypothesis we added *E. coli* (MOI 1000) to the supernatant of Moss55 cells, which constitutively secrete actin. This resulted in a 96% depletion of actin in the supernatant fraction after a 2 hr incubation period (Fig. 3D), confirming that actin is removed from circulation in the mosquito hemolymph by binding to bacteria. In contrast to actin, the binding partner AgMDL1 was continuously secreted into the hemolymph, and its levels decreased to approximately 75% in the presence of both LPS and *E. coli*, as determined by densitometry of western blots, likely because of sequestration by these two immune elicitors as has been shown for a *D. melanogaster* MD2-related protein (Fig. 3C) [21].

### Actin is an extracellular phagocytic factor

**Fig 4:**
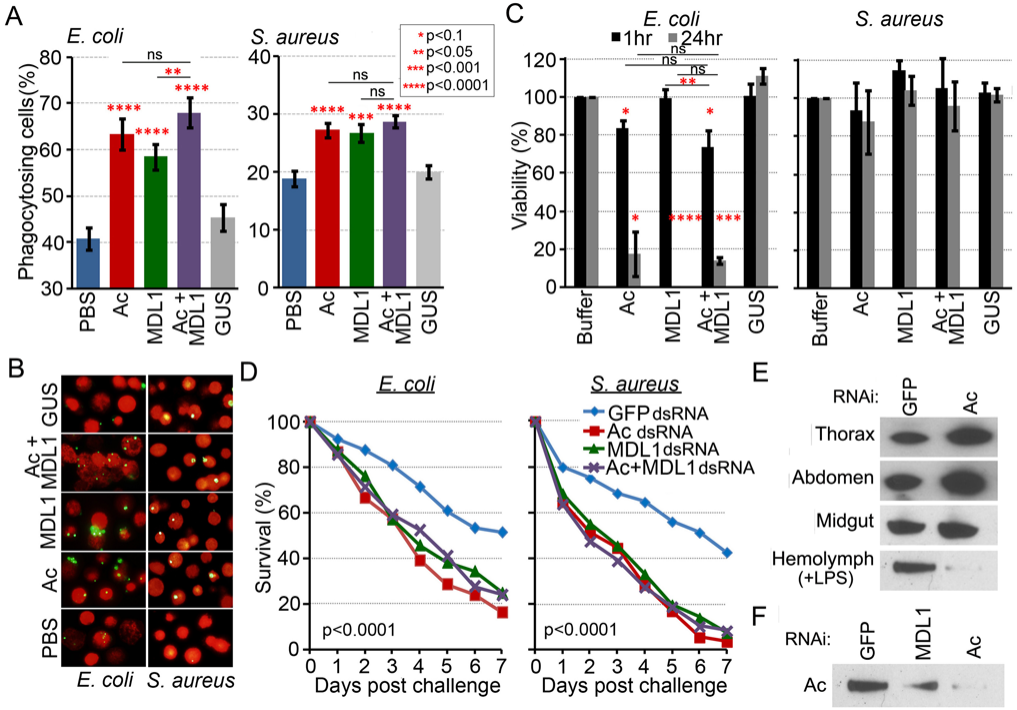
Actin is an extracellular immune factor. (A) Incubation of *E. coli* and *S. aureus* with actin (Ac), AgMDL1 (MDL1), actin +AgMDL1 (Ac+MDL1) or the control protein GUS. Untreated bacteria were incubated with PBS. The percentage of phagocytosing cells was calculated as the number of S2 cells containing at least one phagocytized FITC-labeled bacterium compared to the total number of cells in the field. For each experiment at least 16 fields were counted and the data are representative of three independent experiments. Each bar represents the mean ± the standard deviation. Statistical significance was determined using Student’s *t*-test.(B) Fluorescent microscopy of S2 cells (red) and FITC-labeled *E. coli* or *S. aureus* bacteria (green) incubated with the recombinant proteins actin (Ac) or AgMDL1 (MDL1) alone, or both together (Ac+MDL1) or the control protein GUS. Untreated bacteria were incubated with PBS. (C) Viability of *E. coli* and *S. aureus* was determined after 1 or 24 hr of incubation with recombinant proteins (Ac, MDL1, Ac+MDL1, GUS) and compared to untreated bacteria. Error bars represent the mean ± the standard deviation. Statistical significance was determined using Student’s *t*-test. (D) *An. gambiae* survival rates after silencing of *actin*, *AgMDL1*, *actin* and *AgMDL1*, or *GFP* (control) and challenge of female mosquitoes with *E. coli* (OD 1.5) or *S. aureus* (OD 0.4) four days later. Three biological experiments were performed and combined and statistical analysis consisted of a log-rank test to determine the overall significance between all groups, followed by pairwise comparisons between *GFP* and the other three groups. (E) *An. gambiae* thorax (6 μg), abdomen (6 μg) and midgut (12 μg) tissues along with hemolymph (2 μg) fractions challenged with LPS (100ng) for 4 hr analyzed for the presence of actin 4 days after silencing of *actin* or *GFP* (control). (F) Hemolymph extract from adult female *An. gambiae* probed for the presence of actin after silencing of *GFP* (control), *actin* (Ac) or *AgMDL1* (MDL1).

Our data lead us to hypothesize that actin plays a role in antibacterial defense by acting as a phagocytic or antibacterial factor. To determine whether recombinant actin 5C, alone or in combination with AgMDL1, could influence the phagocytosis of bacteria, we utilized the *D. melanogaster* S2 cell line because its phagocytic properties and assays have been well characterized and established [36, 37]. The presence of actin 5C or AgMDL1 (8μg/200μL) enhanced the percentage of phagocytosing cells, defined as the number of S2 cells containing at least one fluorescein-conjugated *E. coli* compared to the total number of S2 cells in a field, by 14% and 15%, respectively, and *S. aureus* by 14% for both treatments (Fig. 4A, B). For *E. coli*, when both proteins were present, a statistically significant increase in the percentage of phagocytosing cells was detected compared to incubation with AgMDL1 alone. Actin and AgMDL1 together marginally enhanced the percentage of phagocytosing cells compared to actin alone but this effect was not statistically significant. For *S. aureus*, the presence of both proteins did not lead to significant increase in the percentage of phagocytosing cells compared to incubation with individual proteins (Fig. 4A, B). The lack of a significant additive effect on phagocytosis when both recombinant proteins were present was most likely due to the existence of naïve S2 cell-produced actin and MD2 proteins in the medium. These findings demonstrate for the first time that a cytoplasmic actin can enhance phagocytosis of a Gram negative bacterium as an extracellular bacteria-binding immune factor.

### Extracellular actin mediates specific direct antibacterial activity, which is augmented in the presence of AgMDL1

We next investigated the possibility of actin mediating a direct antimicrobial activity through bacterial exposure and subsequent growth assays on a LB agar medium. A 1 hr incubation of *E. coli* with both recombinant actin 5C and AgMDL1 (4μg/100μL) resulted in a 26% reduction in *E. coli* viability, while incubation with actin alone resulted in a 16% reduction (Fig. 4C). After 24 hr incubation, the *E. coli* viability was reduced by more than 80% in both cases. Incubation of *E. coli* with AgMDL1 alone resulted in a 100% inhibition only after a 24-hr incubation, while no antibacterial activity was observed after a 1 hr incubation. However there was a statistically significant difference in viability at the 1 hr time period when comparing bacterial exposure to AgMDL1 alone to co-incubation with both proteins. Interestingly, neither actin 5C nor AgMDL1 had any effect on the viability of *S. aureus*, regardless of the duration of exposure.

### Extracellular actin enhances mosquito survival upon systemic bacterial infection

We have previously shown, through RNA interference (RNAi) assays, that AgMDL1 is involved in antibacterial defense [17]. To provide more physiologically relevant evidence for actin’s role in antibacterial defense, we also investigated the impact of its RNAi-mediated depletion on the survival of adult female mosquitoes after bacterial challenge. Depletion of actin, by targeting *actin 5C* transcripts, compromised the adult mosquitoes’ ability to survive an infection caused by injection of either 10^5^ *E. coli* or 2.7 × 10^4^ *S. aureus* into the mosquito hemolymph at 4 days after double stranded RNA (dsRNA) treatment (Fig. 4D). Co-silencing of *actin* and *AgMDL1* did not result in any additive effect. Since specific silencing of the *actin 5C* gene is difficult because of its high sequence identity with other *actin* family members, we cannot rule out the contribution of other actins to the gene silencing phenotype. Nevertheless, RNAi-mediated depletion of actin did not have any adverse effect on mosquito longevity in the absence of immune challenge (S3 Fig.). Analysis of gene silencing efficiency revealed <30% silencing of muscle actin transcripts, as compared to >75% silencing for the transcripts of the two most abundant cytoplasmic actins (651A and B) (S1 Table). As previously mentioned the biological roles of actin genes are regulated by their expression patterns, which we show as being quite distinct. Thus actin present in the hemolymph fraction only represents a subset of the actin that is present in the cells, and this is the pool of actin that appears to be predominantly affected by RNAi-mediated gene silencing.

*Actin* silencing 4 days prior to 100ng LPS injection resulted in a 96% reduction of actin present in the hemolymph- compared to the GFP dsRNA treated control mosquitoes, while it did not affect the amount of actin detected in the thorax, abdomen and midgut tissues (Fig. 4E, S1 Table). These data, taken together with our bacterial infection and immune challenge-responsive actin transcriptional and proteomic data, suggest that externalized cytoplasmic actin is responsible for mediating mosquito survival upon bacterial infection. Interestingly, depletion of *AgMDL1* transcripts by RNAi resulted in a >50% reduction of actin detected in the hemolymph after LPS challenge, thereby further indicating a functional relevance for the actin–AgMDL1 complex in the mosquito hemolymph (Fig. 4F).

### Actin is a *Plasmodium* antagonist

**Fig 5:**
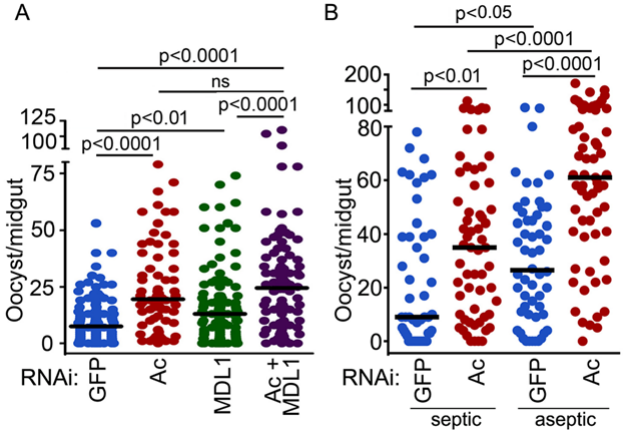
Actin is an antagonist of *Plasmodium* infection. *P. falciparum* oocyst–stage infection intensity after silencing of (A) *An. gambiae actin (Ac)*, *AgMDL1* (MDL1), *actin* and *AgMDL1* (Ac+MDL1), or GFP or (B) *actin* (Ac) or *GFP* in septic vs aseptic mosquitoes. Circles represents the number of oocysts in an individual mosquito midgut, and the horizontal line indicates the median number. Three independent replicates were obtained, and the Mann-Whitney test was used to determine statistical significance and p-values (indicated above each group).

AgMDL1 is an antagonist of *Plasmodium falciparum* infection in the mosquito midgut [17]. Silencing of *actin* alone, or in combination with *AgMDL1*, through dsRNA treatment 4 days prior to feeding on a parasite gametocyte culture resulted in a 2.7- and 3.4-fold increased *P. falciparum* infection intensity, respectively, as a measure of oocyst-stage parasites on the mosquito midgut tissue at 7 days post gametocyte ingestion (Fig. 5A, S2 Table). We and others have shown that the mosquito midgut microbiota can influence susceptibility to *Plasmodium* infection [38] and actin exerts antibacterial activity. To test whether the effect of actin silencing on *P. falciparum* infection was related to the bacteria present in the mosquito midgut, we compared the effect of *actin* silencing on *P. falciparum* infection in antibiotic treated and non-treated mosquitoes (Fig. 5B, S3 Table). Our data show that actin is an antagonist of *P. falciparum* infection regardless of the presence of the mosquito midgut microbiota. This result suggests that actin somehow inhibits the ookinete stage while invading the midgut epithelium or the development of oocysts on the basal side of the midgut.

### Globular and filamentous actins display different immune functions

**Fig 6:**
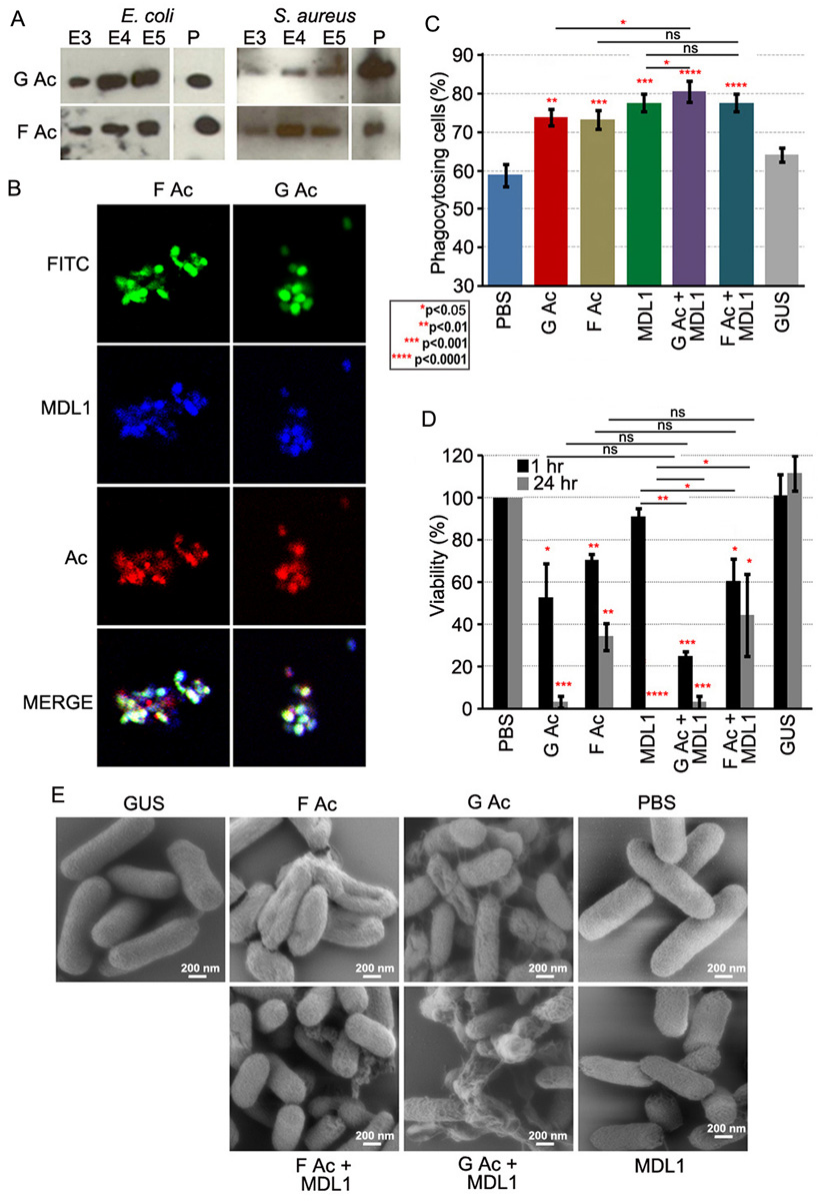
Globular and filamentous actin display different immune properties. (A) Actin content in 0.3–0.5M NaCl eluates (E3–E5 fractions) or pellets (P) of *E. coli* and *S. aureus* incubated with recombinant globular (G Ac) or filamentous (F Ac) actin. (B) FITC-labeled *E. coli* (green) incubated with LPS-stimulated Sua5B cell supernatants and stained for AgMDL1 (blue) and actin (red). Co-localization is indicated in white. (C) The percentage of phagocytosing S2 cells containing at least one *E. coli* bacterium incubated with recombinant globular (G Ac) or filamentous (F Ac) actin with or without AgMDL1 or the control protein GUS as compared to untreated bacteria (PBS). For each assay, at least 16 fields were counted, and the data are representative of three independent experiments. Each bar represents the mean ± the standard deviation. Statistical significance was determined using Student’s *t*-test. (D) Viability of *E. coli* was determined after 1- or 24-hr incubations with recombinant globular (G Ac) or filamentous actin (F Ac), with or without AgMDL1, or the control protein GUS as compared to untreated bacteria (PBS). Error bars represent the mean ± the standard deviation. Statistical significance was determined using Student’s *t*-test. (E) Scanning electron microscopy image of *E. coli* cells after incubation with G actin (G Ac), F actin (F Ac), AgMDL1 (MDL1) alone or together (G Ac+MDL1, F Ac+MDL1) or with the control protein GUS. Untreated cells were incubated with PBS. Scale bar 200 nm.

Because actins can exist in functionally distinct globular and filamentous forms we wanted to investigate whether these two forms also displayed specificity with regards to immune functions. Both globular and filamentous actin bound to *E. coli* and *S. aureus* (Fig. 6A, B) and enhanced phagocytosis of *E. coli* by S2 cells. A more profound phagocytic activity was again seen in the presence of AgMDL1 (Fig. 6C). Incubation of *E. coli* with both G actin and AgMDL1 together resulted in a statistically significant increase in the percentage of phagocytosing cells when compared to incubation of *E. coli* individually with G actin or AgMDL1. For the phagocytic and bacteriocidal assays we focused on the Gram-negative bacterium *E. coli* because actin’s activity against this bacterium was most striking. However, a 1 hr co-incubation of *E. coli* with filamentous actin only reduced the viability of bacteria by 39% in the presence of AgMDL1 and by 30% in its absence, as compared to incubation with globular actin, which reduced the viability by 75% in the presence of AgMDL1, and by 48% in its absence (Fig. 6D). Consistent with what has previously been observed when bacteria are treated with antibacterial factors [39], scanning electron microscopy of *E. coli* after incubation with both forms of actin resulted in a less smooth appearance of the bacterial surfaces (Fig. 6E). Furthermore, in agreement with the antibacterial activity assays, treatment with globular actin together with AgMDL1 resulted in the most pronounced surface irregularities (Fig. 6E). These data suggest functional diversification of the two actin forms.

## Discussion

Here we demonstrate the functional significance of an immune challenge-induced secretion of cytoplasmic actin by insect cells and adult mosquitoes. Extracellular actin forms a complex with the MD2 homolog AgMDL1 and mediates phagocytosis and direct killing of bacteria to which the complex binds with high affinity. Regulation of actin secretion exhibits a certain degree of specificity. Both the Toll and Imd immune signaling pathways can induce actin secretion by immune competent cells, but only exposure of cells to preparations of the major Gram-negative bacteria-derived immune elicitors LPS- and DAP-PGN induced actin secretion, while challenge with the Gram-positive bacteria-specific Lys-PGN does not. While DAP-PGN is a known potent immune elicitor, and IMD pathway activator, in *D. melanogaster*, LPS has not been shown to possess these types of activities. In fact, immune elicitation derived from LPS preparations have been shown to be attributed to contaminating PGN [27, 40], and it is therefore likely that actin externalization upon stimulation with our LPS preparations also derived from contaminating PGN and not the LPS itself. However actin can bind to the surface of both Gram-positive Gram-negative bacteria, and mediate phagocytosis of both *E. coli* and *S. aureus*. It is quite likely that Gram-positive bacteria surface antigens, other than DAP-PGN, induce actin secretion and mediate actin binding. Actin also exerts direct antibacterial activity with some degree of specificity since it only affected the viability of *E. coli* but not *S. aureus*. The physiological relevance of these findings is corroborated by the fact that actin appears to protect mosquitoes against infection with both *E. coli* and *S. aureus* since actin-depleted mosquitoes displayed increased mortality upon challenge with these bacteria. The immune-related actin 5C, which shows higher expression in adult mosquitoes compared to other actins, does not seem to be essential for mosquito survival in a non-challenged context since its depletion in naïve mosquitoes did not compromise longevity.

Previous reports have shown that exposed actin filaments, rather than globular actin, are the ligand for a C-type lectin receptor that detects damaged cells [4, 5] and that actin filaments respond to microbial signals in plants [41]. Our study also shows that globular and filamentous forms of actin display some degree of functional specificity. While both forms can bind to the surface of *E. coli* and *S. aureus* and mediate phagocytosis of *E. coli* to the same degree, the direct bacteriocidal activity was more profound for globular actin, possibly because of a different interaction with the bacterial surface. Globular actin may be better suited for insertion into the bacteria cell wall where it may exert its antibacterial activity. We cannot rule out the possibility that filamentous actin depolymerizes to some extent upon interaction with bacteria, thereby releasing globular actin that can mediate bactericidal activity. Mosquito actin also plays a role as a *Plasmodium* antagonist, and its anti-*Plasmodium* activity is independent of the midgut microbiota which is known to influence susceptibility to the parasite. The role of the mosquito MD2 homolog, AgMDL1, in actin’s immune-related functions is enigmatic. While AgMDL1 augments actin’s interaction with bacterial surfaces, both proteins appear to exert antibacterial properties independently of each other, and the presence of both proteins has only a weak additive effect on phagocytosis but a more profound effect on direct bacteriocidal activity. The lack of a significant additive effect on phagocytosis could be explained by the likely presence of naïve MD2 and actin in the S2 cell culture medium, or alternatively, that an actin-AgMDL1 complex is not necessary for this defense mechanism. The additive effect of AgMDL1 on globular actin-mediated bacteriocidal activity was however quite profound, suggesting some type of cooperation in this defense mechanism. Unlike AgMDL1 which is constitutively secreted into the mosquito hemolymph, actin is only detected upon immune challenge and is considerably reduced upon depletion of AgMDL1. This may suggest that AgMDL1 stabilizes and sequesters actin in the mosquito hemolymph upon its immune challenge-induced secretion, allowing both proteins to exert their anti-pathogen effects. We cannot not rule out the possibility that other AgMDL family members [17] also interact with actin, and keep it in circulation in the mosquito hemolymph. Therefore, complete depletion of hemolymph actin is not achieved after RNAi mediated silencing of *AgMDL1*.

Actin’s anti-*Plasmodium* activity is intriguing although the mechanism by which it inhibits parasite development is unknown and will require further investigation. Phagocytosis has not been implicated in parasite killing at this stage of infection. One can speculate that actin may directly facilitate the destruction of the *Plasmodium* ookinetes as they invade the midgut tissue. The potent anti-*Plasmodium* factor Tep1 is also a phagocytic factor, indicating that certain mechanistic aspects of ookinete killing in the mosquito gut and phagocytosis of bacteria are shared [16, 24]. Previous work has shown that dying ookinetes are surrounded by a polymerized actin zone [42] which is formed from an extension of healthy midgut cells surrounding the parasite, and its synthesis is partially controlled by the transmembrane receptor frizzled-2 [42]. Recent work has also demonstrated that invading *Plasmodium* ookinetes are surrounded by an actin rich structure in the mosquito termed the ookinete hood [43]. The vertebrate AgMDL1 homolog MD2, forms a complex with the TLR4 receptor to interact with LPS and activate immune signaling. The *D. melanogaster* MD2-related proteins NPC2a and NPC2e have been shown to bind to LPS and mediate activation of the Immune Deficiency (Imd) pathway which regulates anti-*Plasmodium* defense in mosquitoes (Shi *et al*., 2012; Clayton *et al*., 2014). In human macrophages the induction of WNT5A, a ligand for frizzled-5 receptors, is dependent on TLR signaling [44]. One can speculate that LPS-mediated externalization of actin and the formation of actin zones / ookinete hoods involve signaling cascades comprising AgMDL1 and frizzled-2 receptors. However, further studies are required to elucidate the regulation of actin externalization and its role as a *Plasmodium* antagonist.

Numerous innate immunity genes have been shown to also have non-immunity functions in processes such as neuronal function and development [45, 46]. Furthermore, other highly abundant proteins, previously considered exclusively intracellular housekeeping factors, have also been found to play roles as extracellular immune factors. Histones, for example, act as receptors for viruses and bacteria at the cell surface, are components of extracellular traps, and exert direct bacteriocidal activity [47]. The addition of actin, another highly expressed versatile intracellular protein, to the repertoire of extracellular immune factors may point to a previously unrecognized facet of immunity in terms of resource management through protein utility. The use of actins, histones, and other vastly abundant and constitutively expressed proteins as immune effectors can be advantageous to cells, because they are immediately available in large quantities upon infection, without taxing the cell to produce proteins that otherwise are not utilized.

## Materials and Methods

### Ethics statement

This study was carried out in strict accordance with the recommendations in the Guide for the Care and Use of Laboratory Animals of the National Institutes of Health. Mice were only used for mosquito rearing as a blood source according to approved protocol. The protocol was approved by the Animal Care and Use Committee of the Johns Hopkins University (Permit Number: M006H300). Commercial anonymous human blood was used for *Plasmodium* infection assays in mosquitoes, and informed consent was therefore not applicable. The Johns Hopkins School of Public Health Ethics Committee has approved this protocol.

### Yeast two-hybrid screen

AgMDL1 was amplified from Sua5B cDNA and cloned into the pGBKT7 bait vector using the primers described in S1 Table according to the Matchmaker Gold Yeast Two Hybrid System user protocol (Clontech). For cDNA library construction, total RNA was isolated from Sua5B cells, reverse-transcribed into cDNA, and cloned into the pGADT7 prey vector using the Make Your Own Mate and Plate Library System (Clontech). pGBKT7 (bait) and pGADT7 (prey) vectors were transformed in the Y2HGold and Y187 yeast strains, respectively. Screening was performed according to the Matchmaker Gold Yeast Two-Hybrid System user protocol (Clontech) using either double dropout synthetic medium (leucine and tryptophan) or quadruple dropout synthetic medium (leucine, tryptophan, adenine, histidine) supplemented with aureobasidin and X-α-Gal (Clontech). Positive clones were sequenced and blasted against the *An. gambiae* genome in Vectorbase. Interacting proteins were confirmed using standard pulldown assays.

### Recombinant protein preparation

Actin 5C and AgMDL1 lacking its endogenous signal peptide were PCR-amplified from *An. gambiae* mosquito cDNA and cloned into the insect expression plasmid pIEX10 (Novagen), using the primers listed in S1 Table. SF9 cells were plated into a 6-well dish at approximately 90% confluency and allowed to attach for 30 min, after which the medium was removed and replaced with 800 μL of SF 900 III serum-free medium (SFM). DNA (1 μg) was mixed with 6 μL of Escort IV transfection reagent (Sigma) and 200 μL of SFM and incubated at room temperature for 30 min, then added dropwise to the cells. At 3 days post-transfection, AgMDL1 was purified from conditioned supernatant, which was centrifuged at 2000 rpm for 10 min to remove cellular debris and then diluted with binding buffer (100 mM Tris-HCl, pH 7.6, with 150 mM NaCl and 0.1% Triton X-100). Actin 5C was obtained from the soluble lysate by lysing cells in hypotonic buffer (5 mM Tris-HCl, pH 7.6, with 0.1 mM MgCl_2_, 1 mM DTT, and EDTA-free protease inhibitors), freeze-thawing the cells twice, then centrifuging the lysate at 14,000 rpm for 30 min at 4°C. The supernatant fraction was retained and diluted in binding buffer. Recombinant AgMDL1 and actin 5C were purified on a Strep II tag column (GE Healthcare) according to the manufacturer’s instructions, then concentrated using a 10-kDa Amicon Ultra filter (Millipore). Globular actin was maintained by incubation in G buffer (5 mM Tris-HCl, pH 7.6, with 0.2 mM CaCl_2_, 0.2 mM ATP, and 0.5 mM DTT). Filamentous actin was formed by adding 10X F actin polymerizing buffer (50 mM KCl, 2 mM MgCl_2_, 1 mM ATP) to G buffer and leaving the recombinant protein mixture at room temperature for 45 min. Recombinant protein was then centrifuged at 14,000 rpm for 2 hr and supernatant fraction retained. Pellets were resuspended in an equivalent volume of water, left on ice for 10 min and equal volumes of supernatant and pellet fractions analyzed by comassie which showed more than 90% polymerization of actin.

### Western blot analysis

Reduced samples were prepared in 5X Laemmli buffer (62.5 mM Tris-HCl, pH 6.8, with 2% SDS, 20% glycerol, 5% β-mercaptoethanol, and 0.01% bromophenol blue), heated at 70°C for 5 min, and separated on a 4–20% gradient gel. Proteins were transferred to nitrocellulose membranes and incubated in blocking buffer (1X PBS with 0.1% Tween-20, 5% BSA) for 1 hr. Blots were incubated for 2 hr in primary antibodies diluted in PBS-T, washed five times with PBS-T, then incubated in secondary antibodies diluted in PBS-T for 1 hr. Membranes were washed five times with PBS-T, and blots were developed using ECL Prime western blotting detection reagent (GE Healthcare). All incubations were performed at room temperature. Mouse β-actin (1:5000) and rabbit α-tubulin (1:3000) were obtained from Abcam. A rabbit polyclonal anti-AgMDL1 antibody (Washington Biotechnology, Inc) was produced using recombinant AgMDL1 protein as described above. Following affinity purification, the antibody was verified by western blot analysis of mosquito hemolymph (1:1000). Anti-rabbit (1:30,000) and anti-mouse (1:30,000) secondary antibodies conjugated to peroxidase were obtained from Jackson Laboratories. Densitometric analysis was performed using Image J software (NIH).

### His tag pull-down assay

Cobalt beads (40 μL; Thermo Scientific) were washed with His buffer (50 mM Tris-HCl, pH 7.6, with 300 mM NaCl, 10 mM imidazole, and EDTA-free protease inhibitors), and incubated at 4°C for 2 hr with 20 μg of recombinant His-tagged AgMDL1 protein. His-tagged GUS was used as a negative control. The slurry was washed three times with 600 μL of buffer to remove excess protein. Sua5B cells were lysed in His buffer, freeze-thawed twice, and centrifuged at 14,000 rpm for 30 min; 200 μL of the soluble fraction (3.5 μg/μL total protein concentration) was retained and incubated overnight with the beads. The next day, the slurry was washed three times with 600 μL buffer. Proteins were eluted with 40 μL elution buffer (50 mM Tris- HCl, pH 7.6, with 300 mM NaCl, 250 mM imidazole, and EDTA-free protease inhibitors); 4 μL of input and 20 μL of eluted fractions were analyzed by western blotting.

### Developmental, tissue and infection-responsive expression analyses

For the analysis of actin transcript abundance in adult female tissues, 3- to 4-day-old mosquitoes were dissected, and the midgut, thorax, and abdomen tissues were removed and placed in Trizol. To measure the transcript abundance of actins in challenged Sua5B cells, 1×10^5^ Sua5B cells were seeded onto a 24-well plate in 0.5 mL of Schneider’s medium and left for 24 hr. The next day, the cells were washed twice with SFM containing no antibiotics and incubated with LPS (10 μg/mL), Lys-PGN (20 μg/mL), DAP-PGN (1 μg/mL) kindly provided by Dr. Neil Silverman, *E. coli*, or *S. aureus* (MOI of 100) for the stipulated time period at 27°C in SFM containing no antibiotics. Total RNA was extracted from mosquito tissues or Sua5B cells using Trizol according to the manufacturer’s protocol. Total cDNA was produced using an oligo-dTprimer and MMLV reverse transcriptase (Promega). SYBR Green PCR mix (Applied Biosystems) was used for real-time quantification of transcripts with an ABI Prism 7300. The ribosomal S7 gene was used for normalization of all cDNA transcripts, and all primers are listed in S1 Table.

### Preparation of insect cell lines supernatant and lysate soluble fractions

Sua5B (*An. gambiae*), Moss55 (*An. gambiae*), MSQ43 (*An. stephensi*), Aag-2 (*Ae. aegypti*) and S2 (*D. melanogaster*) cells were maintained at 27°C in Schneider’s complete medium containing 10% FBS and 1X penicillin/streptomycin. C6/36 (*Ae. albopictus*) cells were cultured in MEM supplemented with 10% FBS, 1% L-glutamine, 1% MEM non-essential amino acids, and 1X penicillin/streptomycin and maintained at 32°C with 5% CO_2._ Cells were seeded at 90% confluency in 2 mL of complete medium in a 6-well plate and left overnight. cells were washed twice with Schneider’s or MEM SFM; 1 mL of Schneider’s or MEM SFM medium was added to each well, and the cells were incubated with LPS (10 μg/mL; *Pseudomonas aeruginosa* serotype 10, Sigma; *E. coli* K12, Ultra-pure Invivogen; *E.coli* O11:B4, Sigma) or peptidoglycan (lys-PGN 20 μg/mL; Staphylococcus aureus or DAP-PGN 1 μg/mL; *E.coli* 1106) for 24 hr. The supernatant fractions were collected and centrifuged at 2,000 rpm for 10 min, then concentrated (20X) using 10-kDa Amicon Ultra filters. For the preparation of cytosolic soluble fraction, cells were lysed in 100 μL of hypotonic buffer freeze-thawed twice, then centrifuged at 14,000 rpm for 30 min. The supernatant fraction was retained and used as the soluble lysate. A Bradford assay was performed, and equal amounts of total protein from the supernatant and soluble fractions were analyzed by western blotting. Constitutive secretion of actin was observed for some cell lines upon prolonged culturing reaching high passage numbers. Previous studies have shown that certain cell lines characteristics can change during increasing passage numbers due to various reasons including changes in cell populations [48, 49, 50].

### Exosome inhibition and isolation

We used the nSMase inhibitor GW4869 (Sigma) to disrupt exosome production trigged by ceramide[31]. Sua5B cells were prepared as described above and challenged with LPS or PGN in the presence of 5 μM GW4869 for 24 hr. Supernatant fractions and cell lysates were prepared as described previously. For exosome isolation, LPS- or Lys-PGN-stimulated supernatant fractions (1 mL) were centrifuged at 2,000 rpm for 30 min. Total exosome isolation solution (500 μL; Invitrogen) was added to the supernatant and left overnight at 4°C. The next day, the mixture was centrifuged at 10,000 rpm for 1 hr at 4°C and the pellet resuspended in 25 μL of hypotonic buffer. Equal amounts of protein for each condition were analyzed by western blotting.

### Incubation of Moss55 cells with *E. coli*

Moss55 cells (1×10^5^) were seeded into a 24-well plate in 0.5 mL of Schneider’s medium and left for 24 hr. The next day, the cells were washed twice with serum-free medium containing no antibiotics and left for an additional 24 hr. *E. coli* were grown overnight, washed twice with PBS, and added to Moss55 cells to a final MOI of 1000 for the stipulated time period at 27°C. Supernatant fractions were concentrated as previously described equal amounts of protein per condition were analyzed by western blotting.

### Cell viability assay

Cell viability was determined using the Cell Titer Fluor Cell Viability Assay (Promega) according to the manufacturer’s instructions. In brief, Sua5B, Moss55, MSQ43, Aag-2, C6/36, or S2 cells were plated to 90% confluency in 100μL of complete medium in a 96-well plate (black with clear bottom) and left overnight. The cells were then washed in serum-free medium and incubated with 100 μL of serum-free medium containing LPS (10 μg/mL) Lys-PGN (20 μg/mL) or DAP-PGN (1 μg/mL) for 24 hr. For saponin treated cells, 50, 000 cells were plated in each well in 100μL of complete media as described above. Next day media was removed and cells were incubated in serum-free medium containing different concentrations of saponin (10^−6^–1%) for 5 min. Control cells were incubated in medium alone. The supernatant was removed, 100 μL of Cell Titer Fluor Reagent was added to all wells and mixed briefly, and the mixture was then incubated at 37°C for three hr. Fluorescence was measured using the Safire II plate reader.

### Bacterial binding assay

The various bacterial species were grown to an OD_600_ of 3.2 and 500uL of each species was incubated overnight with 4 mL of LPS-stimulated cell supernatant fraction, as previously described [15]. For assays done with recombinant protein, minor modifications were implemented: Cultures (4 mL each) were grown overnight, washed twice in PBS, and resuspended in PBS to an OD_600_ of 3.2. Bacteria were pelleted and resuspended in 400 μL of 0.2 M NaCl, inactivated with 10% acetic acid for 10 min, then neutralized with 800 μL of 1M Tris-HCl, pH 7.6. Bacteria were washed three times with PBS and resuspended in 700 μL of 10 mMTris-HCl, pH 7.6, then 25 μL of each bacterial species was added to recombinant protein (8 μg), supplemented with 500 μM ATP, 2 mM MgCl_2_, and 100 μM CaCl_2_ to maintain the activity of actin, in a final volume of 200 μL. Bacteria were washed and eluted with 30 μL of increasing concentrations of NaCl (0.1–0.5M). Pellets were then resuspended in 60 μL of water containing 5X SDS and sonicated, and 5 μL were analyzed by western blotting.

### iTRAQ analysis

The bacterial binding assay was performed as described above using *Enterbacter_Z* and soluble lysates from *An. gambiae* larval and adult mosquitoes and the Sua5B cell line. Sterile PBS was used as a control. The 0.5M NaCl-eluted fractions were TCA-precipitated and sent to the Proteomics Core Facility at Johns Hopkins University for 4plex labeling and analysis as described previously [51].

**Mosquito maintenance**. *An. gambiae* (Keele strain) mosquitoes were maintained on a 10% sucrose solution at 27°C and 70% relative humidity with a 12-hr light/dark cycle according to standard procedures.

### RNA interference (RNAi)-mediated gene silencing in mosquitoes and cell lines

dsRNA was synthesized from PCR-amplified products using the HiScribe T7 In Vitro Translation Kit (New England Biolabs). The *GFP* primers [15] and *caspar* and *cactus* primers [52] have been previously described. The actin5C primers are listed in S1 Table. dsRNA (69 nL, 3 μg/μL) was injected into the thorax of 3- to 4-day-old cold-anesthetized female mosquitoes using a nano-injector (Drummond). For double-silencing experiments, 69 nL (3 μg/μL of each dsRNA was used. Gene silencing was verified by qPCR 3–4 days after dsRNA injection. Significance was determined using the Mann-Whitney test. For cell line experiments, silencing was done as previously described [35].

### Mosquito challenge with bacteria and LPS, and hemolymph isolation

Five to ten cold-anesthetized female mosquitoes were injected with 69 nL PBS, 69 nL *E.coli* OD_600_ 2.8 (~ 2.8×10^9^/mL), or 100 ng LPS/mosquito. After 4 hr, hemolymph was collected as previously described, with slight modifications [53]. 10 μL of an anti-coagulant solution consisting of 70% Schneider’s medium and 30% citrate buffer was injected into the thorax, and diluted hemolymph was collected using a sterile pipette tip coated with Sigmacote (Sigma) through an incision made in the abdominal wall. Hemolymph was centrifuged at 2000 rpm for 10 min at 4°C to remove hemocytes. Equal amounts of total protein were used for western blot analysis.

### Bactericidal assay

Bacteria were grown at 37°C in LB media overnight, centrifuged at 10,000 rpm for 2 min, washed twice with PBS, and resuspended in PBS to 10^8^ CFU/mL. They were then diluted 10 times in 100 μL buffer (10 mMTris, with 500 μM ATP, 2mM MgCl_2_, and 100 μM CaCl_2_) containing 8 μg of recombinant actin or MDL1, alone or together, and left at 25°C. GUS was used as a negative control. After 1 and 24 hr, the mixtures were placed on ice and diluted 10- to 10,000-fold in PBS; 10-μL aliquots were plated onto LB agar plates containing 90 μL of PBS and left overnight at 37°C. Colony-forming units were recorded the next day. *E. coli* and *S. aureus* viabilities were recorded as the number of colonies formed on each plate after treatment, compared to untreated bacteria (recorded as 100%), for each of the three replicates.

### Mosquito survival assay

dsRNA (69 nL, 3 μg/μL) was injected into the thorax of 3- to 4-day-old cold-anesthetized female mosquitoes using a nano-injector (Drummond). Mosquitoes were maintained as described above, and dead mosquitoes were counted over a 27 day period. Three replicates were performed and combined with each group containing at least 50 mosquitoes. Statistical analysis was done in R and consisted of a log-rank test to determine the overall significance between the two groups.

### Mosquito challenge with bacteria and *Plasmodium*

Four days after dsRNA treatment, mosquitoes were injected intrathoracically with 69 nLof *E. coli* OD_600_ 1.5(~1.5×10^9^/mL) or 69 nL of *S. aureus* OD_600_ 0.4(~3.2×10^8^/mL) using a nano-injector. After bacterial challenge, mosquitoes were maintained as described above, and dead mosquitoes were counted over a 7-day period. Three replicates were performed and combined, with each group containing 35 mosquitoes. Statistical analysis was done in R and consisted of a log-rank test to determine the overall significance between all groups, followed by pairwise comparisons between GFP and the other three groups. A Bonferroni correction was used to control for multiple comparisons, meaning that we assessed significance at an α of 0.05/3 = 0.16. For *P. falciparum* experiments, mosquitoes were maintained as previously described and fed an infectious blood meal containing NF54 gametocytes at 4 days post-dsRNA treatment. For septic vs aseptic mosquito infections with *Plasmodium*, antibiotic treatment of mosquitoes was done as previously described [15]. Briefly newly emerged adult female mosquitoes were separated into two cohorts. One group was given fresh filter sterilized 10% sucrose solution containing 50 μg gentamicin sulphate (Quality Biological) and 50 units / 50 μg of penicillin-streptomycin (Life Technologies) per mL. After 3 days of antibiotic exposure the efficacy of the treatment was determined by dissecting the midgut from surface sterilized mosquitoes followed by homogenization of the midgut in PBS. Homogenates were plated onto LB agar and plates incubated for 2 days at room temperature to monitor bacterial growth. RNAi mediated gene silencing in 3–4 day old female mosquitoes was done as described above. Sugar containing antibiotics was replaced with plain sterile sugar 24 hr before feeding with an infectious blood meal containing gametocytes which occurred 4 days post dsRNA treatment. The other group was maintained as previously described and both cohorts were fed on the same infectious blood meal. Unfed mosquitoes were removed 24 hr later. Mosquito midguts were dissected 7 days after *P. falciparum* infection and stained with 0.2% mercurochrome, and oocyst numbers per midgut were determined. Prevalence was defined as the number of mosquitoes with at least one oocyst in its gut; p values were determined using the Mann-Whitney test.

### Phagocytic assay

S2 cells were seeded to 80% confluency in 24-well plate and left for 48 hr, then washed twice with PBS. Fluorescein (Molecular Probes) conjugates of *E. coli* and *Staphylococcus aureus*(10^8^ CFU) were independently incubated for 1 hr with PBS buffer alone or with 8μg/200μL recombinant actin, with or without AgMDL1. Experiments were performed as previously reported [23]. Briefly, after incubation with recombinant protein(s), bacteria were washed twice with 0.1 M Tris buffer, added to S2 cells in 0.5 mL of Schneider’s medium to a final MOI of 100, and incubated at room temperature for 30 min with gentle rocking. Ethidium bromide was then added to the cells as quencher, to a final concentration of 150 μg/mL, for 15 min. Cells were washed three times with PBS and resuspended in 100 μL of PBS; 10 μL of this cell suspension was spotted onto a glass slide and cover slipped. Internalized microbes were detected as reported previously [23, 24, 54]. For each assay, at least 16 fields were counted, and the data are representative of three independent experiments.

### Microscopy

Fluorescein (Molecular Probes) conjugates of *E. coli* (10^8^ CFU) were incubated for 1 hr at room temperature with LPS-stimulated Sua5B cell supernatants. Confocal microscopy was done according to previously reported procedures [23]. In brief, cells were subjected to three 0.1M Tris buffer washes followed by fixing in 4% paraformaldehyde for one hour at room temperature. These *E. coli* cells were then washed with PBS for 3 times followed by blocking with 10% goat serum in PBS for 2 h. Thereafter, *E. coli* cells were incubated with anti-actin (mouse) and anti-AgMDL (rabbit) antibody diluted 1:400 in 1% bovine serum albumin/PBS overnight at 4°C. Samples were washed three times in PBS and incubated for 1 h with Alexa 568 (anti-mouse) Alexa 405 (anti-rabbit) (Molecular Probes) diluted 1:500 in 1% bovine serum albumin/PBS. After 3 PBS washes, the *E. coli* cells were resuspended in 10 ul of PBS and 5 μl of the cells were spotted on the slides and mounted in Prolong Antifade kit (Molecular Probes) with cover slips. Ten sequential optical sections of 1 μm each were collected and only one optical section was shown. Cover slips were sealed with nail polish and subjected to a Zeiss 510 system-based confocal microscopy. For scanning electron microscopy 8 μL of *E.coli (*OD_600_ 5.5) were incubated with 8μg of recombinant G or F actin with or without AgMDL1 or GUS for one hour at 4°C. Control *E. coli* were incubated with PBS. Bacteria were washed three times with PBS, resuspended in 10 μL of PBS, added to coverslips coated with Poly-L-lysine then fixed in 2.5% glutaraldehyde in buffer [0.02M sodium cacodylate buffer supplemented with 1mM MgCl_2_] for 1 hr. Bacteria were then washed three times (10 min each) with buffer and fixed in 1% osmium tetroxide in buffer at 4°C for 2 hr. Bacteria were washed twice (5 min each) with water and dehydrated through a graded ethanol series (30%; 50%; 70%; 90%; 100% (3 times)), then washed for 5 min in 100% ethanol:HMDS (1:1) then HMDS twice (5 min each). Samples were placed in a desiccator overnight, mounted on stubs and coated with 20nm gold-palladium coating using and ion sputter coater. Specimens were then observed with a LEO/Zeiss Field-emission scanning electron microscope at the Johns Hopkins School of Medicine Microscopy Facility.

## Supporting Information

S1 Fig Pull down assay with AgMDL1 and actin. His tagged pull-down of recombinant AgMDL1 incubated with *A. gambiae* Sua5B soluble lysate and probed with an actin antibody. Input represents the starting material and GUS was used as a negative control.
(TIF)

S2 Fig Actin is secreted into the cell culture supernatant fraction upon immune challenge. (A) Immune-challenged (LPS-Pa 10 μg/mL or Lys-PGN 20 μg/mL) Sua5B (*An. gambiae)*, Moss55 (*An. gambiae)*, MSQ43 (*An. stephensi*), C6/36 *(Ae. albopictus)*, Aag-2 (*Ae. aegypti*), and S2 (*D. melongaster*) insect cell supernatants and soluble lysate fractions examined for the presence of actin.(B)Viability of all cell lines after treatment with LPS (10 μg/mL), Lys-PGN (20 μg/mL) or DAP-PGN (1 μg/mL) for 24 hr was determined using the Cell Titer Fluor Cell Viability Assay.
(TIF)

S3 Fig Silencing of *actin* does not affect longevity in *An. gambiae* female mosquitoes. *An. gambiae* survival rates of female mosquitoes after silencing of *actin* (*Ac)* or *GFP* (control). Three biological experiments were performed and combined and statistical analysis consisted of a log-rank test to determine the overall significance between the two groups.
(TIF)

S1 Table PCR primers used for the yeast-two-hybrid screen, recombinant protein, RNAi, and qRT-PCR. (DOCX)

S2 Table Infection data (*Plasmodium* parasite numbers) for gene-silenced mosquitoes. (DOCX)

S3 Table Infection data (*Plasmodium* parasite numbers) for septic vs aseptic *actin* silenced mosquitoes. (DOCX)
