# Supplementary figures and images for: Cytoplasmic Actin Is an Extracellular Insect Immune Factor which Is Secreted upon Immune Challenge and Mediates Phagocytosis and Direct Killing of Bacteria, and Is a Plasmodium Antagonist
Source: PLoS Pathog. 2015 Feb 6;11(2):e1004631. doi: 10.1371/journal.ppat.1004631 (PMC4450071; doi:10.1371/journal.ppat.1004631)

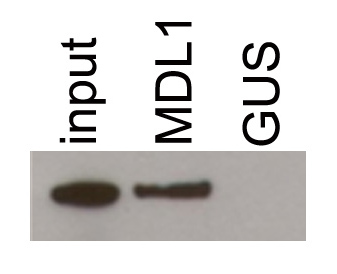

Supplement: S1 Fig — His tagged pull-down of recombinant AgMDL1 incubated with A. gambiae Sua5B soluble lysate and probed with an actin antibody. Input represents the starting material and GUS was used as a negative control. (TIF) [file ppat.1004631.s001.tif]

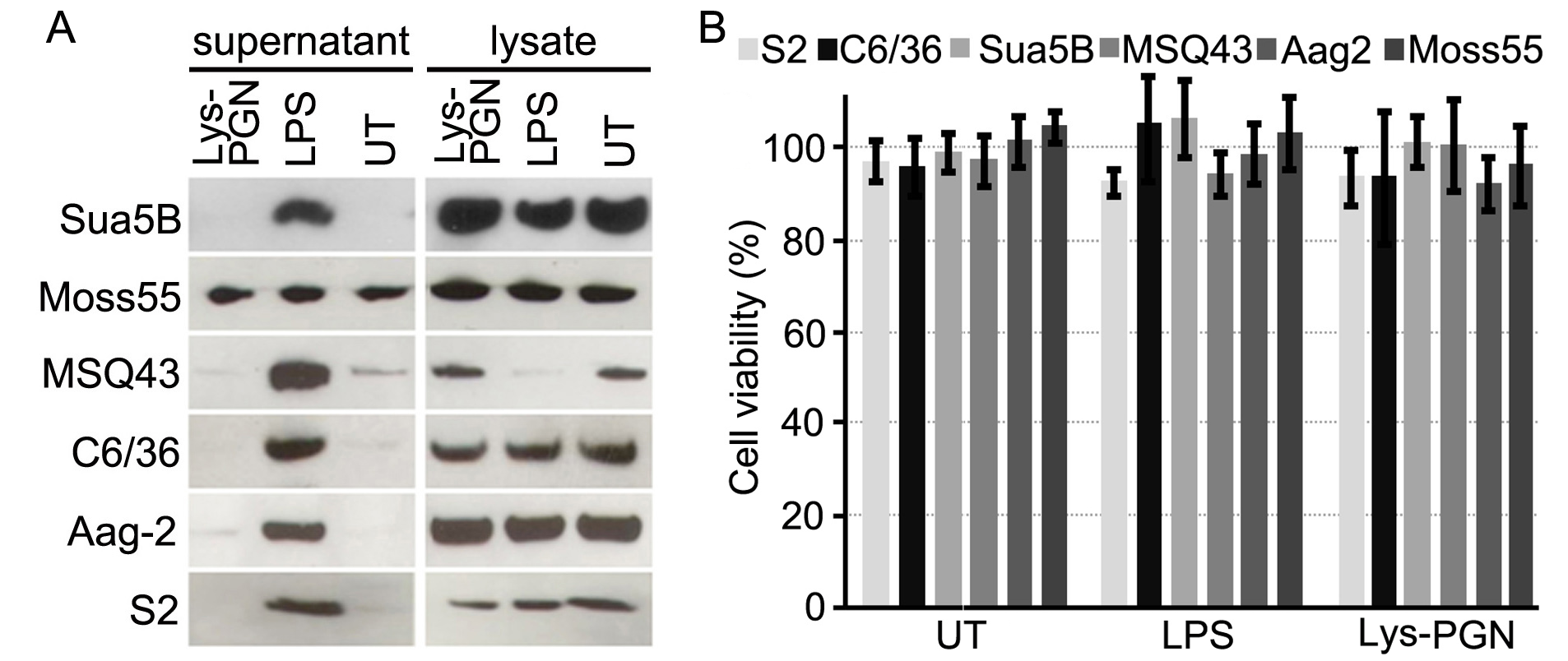

Supplement: S2 Fig — (A) Immune-challenged (LPS-Pa 10 μg/mL or Lys-PGN 20 μg/mL) Sua5B (An. gambiae), Moss55 (An. gambiae), MSQ43 (An. stephensi), C6/36 (Ae. albopictus), Aag-2 (Ae. aegypti), and S2 (D. melongaster) insect cell supernatants and soluble lysate fractions examined for the presence of actin.(B)Viability of all cell lines after treatment with LPS (10 μg/mL), Lys-PGN (20 μg/mL) or DAP-PGN (1 μg/mL) for 24 hr was determined using the Cell Titer Fluor Cell Viability Assay. (TIF) [file ppat.1004631.s002.tif]

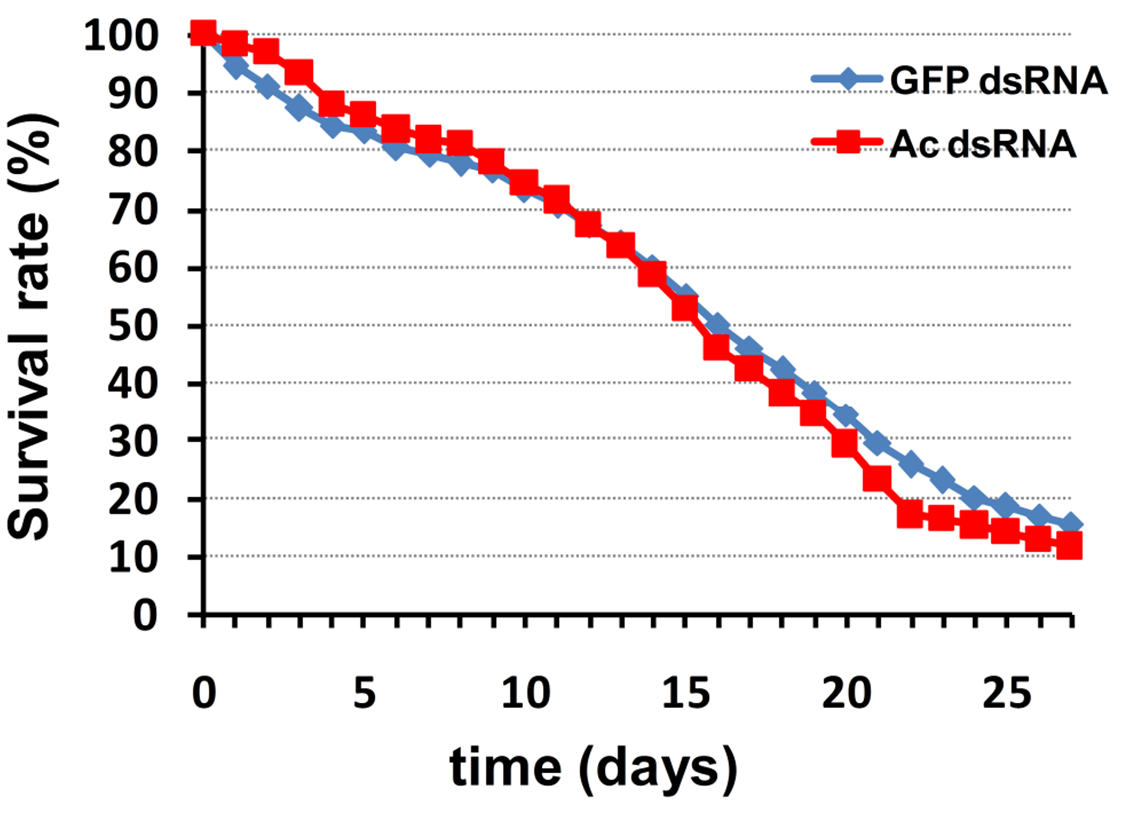

Supplement: S3 Fig — An. gambiae survival rates of female mosquitoes after silencing of actin (Ac) or GFP (control). Three biological experiments were performed and combined and statistical analysis consisted of a log-rank test to determine the overall significance between the two groups. (TIF) [file ppat.1004631.s003.tif]
