# Supplementary material for: Cytoplasmic Actin Is an Extracellular Insect Immune Factor which Is Secreted upon Immune Challenge and Mediates Phagocytosis and Direct Killing of Bacteria, and Is a Plasmodium Antagonist
Source: PLoS Pathog. 2015 Feb 6;11(2):e1004631. doi: 10.1371/journal.ppat.1004631 (PMC4450071; doi:10.1371/journal.ppat.1004631)
Supplement: S1 Table — (DOCX) [file ppat.1004631.s004.docx]

**Supplemental Table S1. PCR primers used for the Y2H screen, recombinant protein, RNAi, and qRT-PCR.**

| **Primer Name** | **Primer sequence** | **KD% (±SE)** |
| --- | --- | --- |
| MDL1 Y2HF  MDL1 Y2HR | CATGGAGGCCGAATTCATGAAGTACTTTCAAACAGTCGC  GCAGGTCGACGGATCCCTAAGTGCGCTTGCTCTTCTTGG |  |
| rMDL1 HisF  rMDL1 HisR | GACGACGACAAGATGGAAGTAGTAAACTTTAAG  GAGGAGAAGCCCGGTGCAGTGCGCTTGCT |  |
| rAc5C HisF  rAc5C HisR | GACGACGACAAGATGTGCGACGAAGAG  GAGGAGAAGCCCGGTGCGAAGCACTTTCG |  |
| Actin RNAiF  Actin RNAiR | TAATACGACTCACTATAGGTGTTCGAGACGTTCAACACA  TAATACGACTCACTATAGGATCGCACTTCATGATCGAGT |  |
| Ac651A qPCRF  Ac651A qPCRR | CTTGCATTAACCGAGTGCTGC  GCCTTGCACATTCCGGATCC | **78(±12)** |
| Ac651B qPCRF  Ac651B qPCRR | TGACTTTGTGAACGCGCACC  GCCTTGCACATTCCGGATCC | **89(±7)** |
| Ac651C qPCRF  Ac651C qPCRR | GCGTATCAAATAAGGCACGCG  GCCTTGCACATTCCGGATCC | **45(±5)** |
| Ac11516 qPCRF  Ac11516 qPCRR | ACAAACAACAAATCCAAAATG  GCCTTGCACATTCCGGATCC | **29(±8)** |
| Ac5095 qPCRF  Ac5095 qPCRR | CCAAAACCAAACCAGCCAAAATG  GCCTTGCACATTCCGGATCC | **75(±13)** |
| Ac1676 qPCRF  Ac1676 qPCRR | CACTCAACAGCAAGCCTCAAG  GCCTTGCACATTCCGGATCC | **67(±12)** |
| Ac2127 qPCRF  Ac2127 qPCRR | TGTGATAGTGAATCAACCGG  CGTGCTTCGGCCGGCCCATG | **81(±14)** |
| MDL1 qPCRF  MDL1 qPCRR | GCCTACGATTTGCCCATCAA  TGGTGATGTTGATCTGCACG | **66(±6)** |
