# Supplementary material for: Cytoplasmic Actin Is an Extracellular Insect Immune Factor which Is Secreted upon Immune Challenge and Mediates Phagocytosis and Direct Killing of Bacteria, and Is a Plasmodium Antagonist
Source: PLoS Pathog. 2015 Feb 6;11(2):e1004631. doi: 10.1371/journal.ppat.1004631 (PMC4450071; doi:10.1371/journal.ppat.1004631)
Supplement: S2 Table — (DOCX) [file ppat.1004631.s005.docx]

**Supplemental Table S2. Infection data (*Plasmodium* parasite numbers) for gene-silenced mosquitoes.**

|  | **GFP** | **MDL1** | **Ac** | **Ac+MDL1** |
| --- | --- | --- | --- | --- |
| **n=** | 108 | 107 | 70 | 92 |
| **Range** | 0-53 | 0-74 | 0-79 | 0-84 |
| **Prevalence** | 87% | 89.7% | 98.5% | 95.6% |
| **Median** | 7 | 13 | 19 | 24 |
| **p -value** |  | 0.0047 | <0.0001 | <0.0001 |
