# Supplementary material for: Cytoplasmic Actin Is an Extracellular Insect Immune Factor which Is Secreted upon Immune Challenge and Mediates Phagocytosis and Direct Killing of Bacteria, and Is a Plasmodium Antagonist
Source: PLoS Pathog. 2015 Feb 6;11(2):e1004631. doi: 10.1371/journal.ppat.1004631 (PMC4450071; doi:10.1371/journal.ppat.1004631)
Supplement: S3 Table — (DOCX) [file ppat.1004631.s006.docx]

**Supplemental Table S3. Infection data (*Plasmodium* parasite numbers) for septic vs aseptic gene-silenced mosquitoes.**

|  | **GFP septic** | **Ac septic** | **GFP aseptic** | **Ac aseptic** |
| --- | --- | --- | --- | --- |
| **n=** | 49 | 60 | 54 | 63 |
| **Range** | 0-72 | 0-113 | 0-91 | 0-171 |
| **Prevalence** | 76% | 93% | 91% | 98% |
| **Median** | 9 | 35 | 26.5 | 61 |
| **p -value** |  | 0.0015 |  | <0.0001 |
